# Naringenin-Grafted Polyvinyl Alcohol (Na/PVA) Nanoparticles: Synthesis, Characterisation and *In Vitro* Evaluation of Its Antimicrobial Efficiency on Fresh Beef

**DOI:** 10.21315/tlsr2022.33.1.9

**Published:** 2022-03-31

**Authors:** Syarifah Ab Rashid, Nur Suhaili Mohamad Rosli, Teo Siew Hway, Tong Woei Yenn, Leong Chean Ring, Fahmi Asyadi Md Yusof, Tan Wen-Nee

**Affiliations:** 1 Universiti Kuala Lumpur, Branch Campus Malaysian Institute of Chemical and Engineering Technology, Lot 1988 Kawasan Perindustrian Bandar Vendor, Taboh Naning, 78000 Alor Gajah, Melaka, Malaysia; 2 Chemistry Section, School of Distance Education, Universiti Sains Malaysia, 11800 USM Pulau Pinang, Malaysia

**Keywords:** Naringenin, Antimicrobial Activity, Nanoparticles, Meat, Naringenin, Aktiviti Antimikrob, Nanozarah, Daging Lembu Segar

## Abstract

Food preservatives are commonly used to guarantee the safety and quality of food. Naturally-derived preservatives are preferred by the consumers as chemical preservatives are associated with adverse side effects. The application of naringenin as a food preservative is restricted due to its high volatility, albeit known for its good efficacy in inhibiting microbial growth on food. Thus, this study aimed to synthesis and characterise naringenin-grafted PVA (Na/PVA) nanoparticles as potential meat preservative. The nano-size of naringenin was characterised by transmission electron microscopy. Finally, the antibacterial efficiency of Na/PVA nanoparticles substance was evaluated on disc diffusion assay, broth microdilution assay and using fresh beef as food model. The naringenin nanoparticles appeared in globule and smooth structure, with an average size of less than 200 nm. In total, 11.08% of naringenin nanoparticles was successfully encapsulated into PVA nanoparticles. Based on Fourier transform infrared spectra, naringenin was successfully encapsulated into PVA nanoparticles. The release of naringenin from Na/PVA nanoparticles was slow and gradual, with an initial burst release effect observed. The property was ideal for application in food model. It gained a plateau at 48 h. The Na/PVA nanoparticles depicted its antibacterial efficiency on all the test foodborne bacteria, except *E. coli*. It was represented by the presence of inhibition zones, which were ranged from 10 mm to 14 mm. The activity was concentration-dependent, and a higher concentration of nanoparticles was needed to kill the test bacteria. The meat coated with Na/PVA nanoparticles displayed a 100% reduction in bacterial load, where no bacterial growth was detected at the sample throughout the incubation period. The coating of nanoparticles also improved the organoleptic property of fresh beef, by maintain the meat colour. The developed nanoparticles can be potentially used nano-preservative to maintain the microbiological quality of fresh beef.

## Abstract (fr)

Pengawet makanan biasanya digunakan untuk menjamin keselamatan dan kualiti makanan. Pengawet semulajadi yang diperoleh ialah pilihan oleh pengguna kerana pengawet kimia dikaitkan dengan kesan sampingan yang buruk. Penggunaan naringenin sebagai pengawet makanan adalah terhad kerana turun naik yang tinggi, walaupun dikenali kerana keberkesanan yang baik dalam menghalang pertumbuhan mikrob pada makanan. Oleh itu, kajian ini bertujuan untuk mensintesis dan mencirikan nanozarah PVA (Na/PVA) naringenin sebagai pengawet daging yang berpotensi. Saiz nano naringenin dicirikan oleh mikroskopi elektron penghantaran. Akhirnya, kecekapan antibakteria bahan nanozarah Na/PVA dinilai pada *disc diffusion assay*, *broth microdilution assay* dan menggunakan daging lembu segar sebagai model kajian. Naringenin nanozarah muncul dalam globul dan struktur licin, dengan saiz purata kurang daripada 200 nm. Secara keseluruhan, 11.08% daripada naringenin nanozarah telah berjaya dimasukkan ke dalam nanozarah PVA. Berdasarkan pengubah spektra inframerah Fourier, naringenin berjaya dikapsulkan dalam nanozarah PVA. Pelepasan naringenin dari Na/PVA nanozarah adalah perlahan dan beransur-ansur, dengan kesan pelepasan mendadak diperhatikan pada fasa awal kajian. Sifat ini sesuai digunakan untuk menghalang pertumbuhan mikrob pada makanan. Ia mendapat dataran tinggi pada 48 h. Nanozarah Na/PVA menggambarkan kecekapan antibakteria pada semua bakteria bawaan makanan ujian, kecuali *E. coli*. Ia diwakili oleh kehadiran zon perencatan, yang berkisar dari 10 mm hingga 14 mm. Aktiviti ini bergantung kepada kepekatan, dan kepekatan nanozarah yang lebih tinggi diperlukan untuk membunuh bakteria ujian. Daging yang disalut dengan Na/PVA nanozarah menunjukkan pengurangan 100% dalam beban bakteria, di mana tiada pertumbuhan bakteria dikesan pada sampel sepanjang tempoh inkubasi. Lapisan nanozarah juga meningkatkan sifat organoleptik daging lembu segar, dengan mengekalkan warna daging. Nanozarah yang dibangunkan berpotensi digunakan nano-pengawet untuk mengekalkan kualiti mikrobiologi daging lembu segar.

**Highlight**

- Naringenin nanoparticles were successfully developed using polyvinyl alcohol as encapsulant.
- Naringenin nanaoparticles exhibited broad spectrum antibacterial activity on both Gram positive and negative bacteria.
- Naringenin nanoparticles reduced the bacterial load on fresh meat samples by 100%.

## INTRODUCTION

Fresh beef is a high-energy type of food with high nutritional value. It has a high content of vitamins and minerals. It also provides an essential source of high-quality protein (Jamilah *et al*. 2008). However, fresh red meat has a short shelf life due to microbial spoilage and lipid oxidation. It reaches its maximum shelf life based on several contributing factors such as pH, temperature, microbial growth and water activity. Microbial growth is the primary factor causing spoilage of fresh red meat, followed by the deterioration of the colour. About 24 to 81 million foodborne illnesses were reported every year, and 50% of these cases were associated with meat and poultry (Ahmed & Sarangi 2013). The shelf life of the meat products is degraded due to microbial contamination during slaughtering and processing. *Stapylococcus aureus*, *Salmonella* sp., *Campylobacter* species, *Listeria monocytogenes* and *Escherichia coli* are frequently associated with animal-based food (Abebe *et al*. 2020). Cross contamination of the animal carcasses with bacteria could occur during slaughtering process (Girma 2015) and may affect the meat quality.

In order to prevent microbial growth in food products, antimicrobial agents are added directly into the product, surface coated or adapted in the packaging material. Today, meat manufacturers are observing for new approaches to lessen the microbial growth in their products. However, the usage of nitrates and nitrites caused further risks to health (Silva & Lidon 2016). These substances react with amines to form carcinogenic nitrosamine. Nitrosamine is a genotoxic carcinogen that leads to cancerous cells and tumours formation. Long-term consumption of chemical additives also causes headaches, stomach-ache and allergies. Despite using synthetic chemical additives, consumers tend to choose food products preserved with natural additives. Meat additives such as sulfur dioxide, ethylene diamine, tetraacetic acid, butylated hydroxyanisole BHA, fumarate esters, sodium hypophosphite are also used as alternatives to nitrate and nitrite for meat preservatives. However, potential adverse health effects may appear over time with the applications of these chemicals (Gassara *et al*. 2016).

Naringenin (IUPA: (2S)-5,7-Dihydroxy-2-(4-hydroxyphenyl)-2,3-dihydro-4H-chromen-4-one) is a flavonoid compound found in citrus fruits, tomatoes, cherries, grapefruit and cocoa. Naringenin exhibits good biological properties as anti-atherogenic, anti-cancer, anti-inflammatory, anti-fibrogenic and antioxidant (Ji *et al*. 2016). Besides, naringenin is a FDA-permitted food flavouring substance. However, the usage in food systems is limited because of poor water solubility, as water is often used as a main raw ingredient in most of the food manufacturing processes. Moreover, the pungent taste and smell could interfere with the protein and fat present in food, which causes low palatability. It is believed that naringenin contributes to the bitterness in some citrus fruits, albeit the addition of naringenin in food could benefit health (Teng, Liu, *et al*. 2018). All these shortcomings limit the usage of naringenin as an antimicrobial agent in the food system.

Nanotechnology is a new technology that is impacting the food and poultry industry. This technology successfully improved the whole food security system, starting from the manufacturing to processing, storage and development of ingenious materials and applications (Ezhilarasi *et al*. 2012). Besides, nanoparticles are also used in food processing, food packaging, functional food development, food safety, detection of foodborne pathogens, and shelf-life extension of food and food products, in order to improve the food security system (Gaba *et al*. 2019). The application of nanotechnology successfully improved the texture, taste, other sensory attributes, colour, stabilities during the storage and shelf life of meat-based products. In this study, we aimed to develop naringenin nanoparticles using polyvinyl alcohol (PVA) as an encapsulant material. The antimicrobial activity of the developed nanoparticles was evaluated on foodborne bacteria and using fresh beef as food model.

## MATERIALS AND METHODS

### Synthesis of Na/PVA

Naringenin nanoparticles were synthesised according to Sahadan *et al. (*2019). A total of 200 μg of naringenin (Alfabiotech, UK) was first dissolved in 5 mL of methanol (Thermo Fisher, USA), followed by the addition of 1 g of Pluronic F127 (Sigma Aldrich, USA). The mixture was homogenised by using a Silent Crusher (Heidolph, Germany) at 10,000 rpm for 5 min in an ice bath. Next, 50 mL of 2% PVA solution (R&M Chemicals) was added into the mixture and agitated at the same agitation speed for 10 min. The transparent solution was kept at −80°C and followed by a freeze-drying process (Labconco, USA). The freeze-dried nanoparticles were kept in a desiccator at room temperature (25 ± 2°C) prior to use. The blank nanoparticles that served as a negative control for antimicrobial assays were synthesised by replacing the naringenin solution with methanol. The nanoparticle powder was dissolved in 30% Tween 20 solution to an appropriate concentration, prior to filtration process via 0.22 μm pore size filter (Millipore, USA).

### Microscopic Observation of Nanoparticles

The structure and morphology of naringenin nanoparticles were visualised under transmission electron microscopy (Philips CM12, Netherlands). A small drop of the nanoparticle solution was placed on a carbon-coated copper grid. Next, uranyl acetate stain was added to the grid. The sample was left to dry in a fume hood chamber, prior to transmission electron microscopy (TEM) observation.

### Encapsulation Efficiency Analysis

The encapsulation efficiency refers to the amount of naringenin encapsulated into PVA nanoparticles (Zhang *et al*. 2014). At first, 10 mg of nanoparticles were added to 10 mL of ethanol. This mixture was exposed to the sonication process for 15 min in order to release the naringenin encapsulated in the nanoparticles. The naringenin content was measured at the absorption of 290 nm by using Flexar FX-20 High-Performance Liquid Chromatography (HPLC, Perkin Elmer, USA). One microlitre of the test sample was transported through the C18 column and separated by a mobile phase containing methanol and 0.2% (v/v) phosphoric acid. The result was detected at a wavelength of 290 nm, with a flow rate of 1.0 mL min^−1^. Naringenin standard was prepared at a concentration range of 15.6 to 250.0 μg mL^−1^. The amount of naringenin present in the sample was estimated according to an equation resulting from the calibration curve. Then, the encapsulation efficiency was measured based on the formulation below.


$$\text{Encapsulation efficiency }(\%)=\frac{\text{Amount of naringenin entrapped}}{\text{Initial amount of naringenin added}}\times 100$$

### Observation of the Functional Groups in Na/PVA Nanoparticles

This analysis was carried out to investigate the chemical interfaces between naringenin and PVA. The spectra of naringenin alone, PVA alone and Na/PVA nanoparticles were analysed through Thermo Scientific Nicolet IS10 (USA). The infrared region was set at a wavenumber of 4,000–400 cm^−1^.

### Naringenin Release Property

Firstly, 100 mg of naringenin nanoparticle was placed in 10 mL phosphate buffer solution (pH 7.4). The solution was shaken in an incubator shaker at 200 rpm, 37°C. At pre-adjusted time points of 1 h, 2 h, 4 h, 8 h, 24 h, 48 h and 96 h, 500 μL of the sample was taken out from the flask. The amount of naringenin released in the test medium was analysed via HPLC as per the protocol described for encapsulation efficiency analysis. The graph of amount of naringenin released against time was plotted to study the naringenin release behaviour of the nanoparticle system.

### Test Bacteria, Source and Maintenance

The antibacterial spectrum of naringenin nanoparticles was inspected against foodborne bacteria. The test bacteria were Gram-positive bacteria (*Bacillus cereus*, *Staphylococcus aureus*, methicillin-resistant *S. aureus* [MRSA]) and Gram-negative bacteria (*Escherichia coli*, *Salmonella typhimurium*, *Yersinia enterocolitica*). These bacteria were previously isolated from contaminated food samples by Leong *et al*. (2017). All test bacteria were cultured on nutrient agar (Merck, USA) fortnightly to sustain their viability. The inoculum concentration of the test bacteria was 1 × 10^8^ CFU mL^−1^. This bacterial suspension was prepared by scratching one loopful of the bacterial colonies and suspended it in 10 mL of sterilised distilled water. After a vigorous, the turbidity was compared to 0.5 Mc Farland standard, prior to use.

### Disc Diffusion Assay

Disc diffusion assay was executed as described by Tong *et al*. (2014) to determine the antimicrobial spectrum of Na/PVA nanoparticles. A total of four test substances were used, including Na/PVA nanoparticles (100 μg mL^−1^), pure naringenin (100 μg mL^−1^), blank nanoparticles (without naringenin, 100 μg mL^−1^) and control (30%; v/v Tween 20). Initially, the bacterial suspension was streaked on the surface of agar (Mueller Hinton agar, Merck, USA) by using a sterilised cotton swab. Then, 20 μL of test Na/PVA nanoparticles substance was pipetted on a sterile paper disc (6 mm diameter) and left to dry. Next, the paper disc was laid on the inoculated Mueller Hinton Agar (MHA) surface by using a sterile forcep. All plates were kept at 37°C for 24 h. After the incubation period, the formation of clear zone surrounding the paper disc was observed and its diameter was estimated using a ruler.

### Broth Microdilution Assay

The minimal inhibitory concentration (MIC) and minimal bactericidal concentration (MBC) of Na/PVA nanoparticles were tested on the test bacteria that exhibited a notable inhibitory activity during disc diffusion assay (*B. cereus*, MRSA, *S. aureus*, *S. thypi* and *Yersinia* sp.). The assay was performed in a flat bottom 96 well plate (Biologix, USA). The inoculum was prepared by adding 1 mL of the bacterial suspension into 9 mL sterile double strength Mueller Hinton broth (MHB, Merck, USA). Then, 100 μL of this bacterial suspension was pipetted into each well. It was followed by 100 μL of Na/PVA nanoparticles substance, which concentrations were prepared at 1.6 to 200 μg mL^−1^. These combinations have provided a final concentration range of 0.8 to 100 μg mL^−1^. The inoculated broth was added with blank nanoparticle dissolved in 30% (v/v) Tween 20 solution for growth control. As for sterility control, the sterile broth was added with Na/PVA nanoparticles at various concentrations (0.8 to 100 μg mL^−1^). The plate was kept at 37°C for 24 h. Then, 20 μL of 0.2 mg mL^−1^ *p*-iodonitrotetrazolium violet salt (Sigma, USA) was pipetted into each well to determine the viability of the test bacteria. Next, the plate was placed in the dark for 30 min and the temperature was set at 37°C. The colour changes (from yellow to pink) designate the viability of the test bacteria. MIC was recorded as the lowest concentration of naringenin nanoparticles that inhibit bacterial growth. The MBC was determined by taking one loopful sample from each well and further streaked on the MHA plate. The plates were kept at 37°C for 24 h and the bacterial growth was monitored. MBC was defined as the lowest concentration of Na/PVA nanoparticles needed to kill the test bacterium.

### Application of Na/PVA Nanoparticles on Meat

The beef meat sample (Australian chilled beef, Topside, Australia) was purchased from a local hypermarket at Pulau Sebang, Melaka, Malaysia. The beef samples were randomly selected within the validity date, collected and aseptically placed in a sterile collection bag. The sample was transported in an icebox at 4°C until further use. Method of FDA (2012) was applied in this section. The antibacterial efficiency was further evaluated according to the bacterial load on the food model. The meat cube was sampled every 24 h. Firstly, 10 g of the meat was soaked for 60 min in Na/PVA nanoparticles substance. Water excess from the meat was dripped for 1 min. After that, it was placed in 100 mL sterile peptone water (Oxoid, UK). Then, it was homogenised in a stomacher (Seward 80, UK) for 1 min. Next, 1 mL of the homogenate was serially diluted (10-fold) with sterile peptone water. One hundred microlitres of the diluents were spread on plate count agar plates by using a sterile spreader. The plates were incubated at 30°C for 48 h. The number of colonies were observed under the colony counter. The experiment was triplicated. The result was presented as a logarithm of the number of the viable cells (CFU mL^−1^) versus incubation time. Meat coated with 30% (v/v) Tween 20 was set as control.

### Statistical Analysis

All tests were performed in three replicates. Data were reported as means ± standard deviation (SD). Results were analysed statistically (*p* < 0.05) by using SPSS (IBM, USA).

## RESULTS

### Na/PVA Nanoparticles Size and Their Encapsulated Efficiency

**Figure 1:**
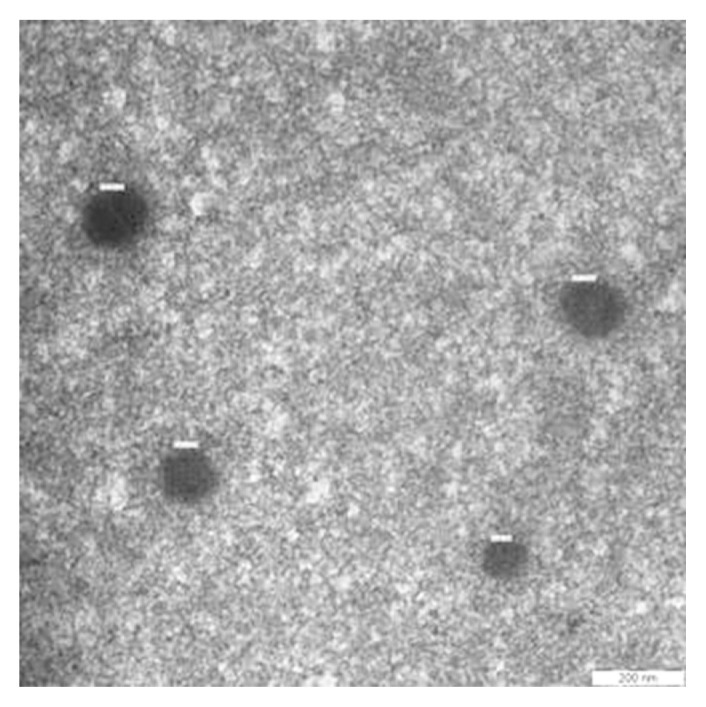
Microscopic observation of Na/PVA nanoparticles in the realm of 200 nm.

TEM was used to transmit a beam of electrons through the ultra-thin Na/PVA nanoparticles to form an image, as displayed in Fig. 1. It denotes the average diameter of the developed nanoparticles was less than 200 nm. The nanoparticles were globules and smooth, with a size range of 92.36 to 127 ± 0.3 nm. Homogeneous and null aggregation structures were visualised. Encapsulation efficiency reflects the percentage of drug entrapped in the polymeric matrix. Therefore, the encapsulation efficiency can be determined by checking the amount of naringenin trapped in the nanoparticles. In this experiment, 11.08% of naringenin was successfully entrapped in PVA nanoparticles.

### Functional Groups in NA/PVA Nanoparticles

**Figure 2:**
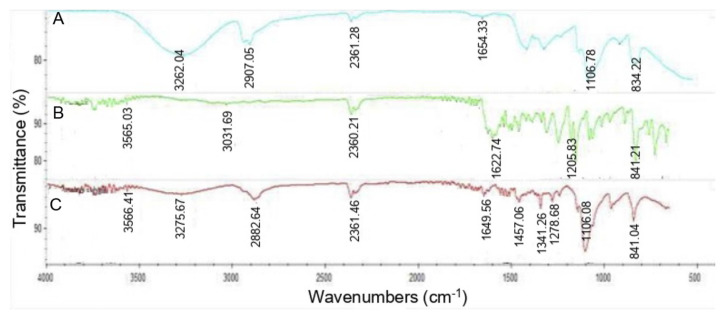
The infrared spectrum for PVA (A), naringenin (B) and Na/PVA nanoparticles (C).

The Fourier transform infrared spectroscopy (FTIR) spectra stipulate the information of crosslinking mechanism and functional groups of the Na/PVA nanoparticles. To interpret the spectra of the Na/PVA sample, two spectrum regions were divided. Regions from 4,000 to 1,300 cm^−1^ are for functional groups and the regions from 1,300 to 400 cm^−1^ are allocated for molecular fingerprint. Fig. 2 signifies the infrared spectra of PVA, naringenin and Na/PVA nanoparticles. The spectrum of PVA has a broad absorption peak of around 3,262 cm^−1^ for stretching vibration of −OH moiety (Alhosseini *et al*. 2012). The event occurs due to intra and extramolecular hydrogen bonding. Tailing spectra were observed in between 2,840 and 400 cm^−1^. An obvious spectrum was developed at wavenumber 2,907 cm^−1^, which represented C-H stretching of alkyl groups. The aromatic groups, which generally related to C=O and C-O stretching moieties, were observed at a wavenumber of 1,654 cm^−1^ (Mansur *et al*. 2008; Wang *et al*. 2004) (Fig. 2A). The infrared characteristics of naringenin have been examined previously by Joshi *et al*. (2018). It resulted in absorption peaks development at 3,286 cm^−1^ and 1,600 cm^−1^. These peaks parallel to the alcoholic (−OH) and keto (C=O) group, respectively. The appearance of small and sharp peaks around 1,600 to 1,500 cm^−1^ represented the aromatic rings. These events are normally followed by other peaks ranging from 3,150 to 3,000 cm^−1^, representing the C-H stretching vibrations (Nandiyanto *et al*. 2019) (Fig. 2B). All these functional groups were successfully detected in Na/PVA nanoparticles spectrum (Fig. 2C). As observed, there were small peaks obtained from 4,000 cm^−1^ to 3,000 cm^−1^, that signified the alcoholic groups (−OH stretching). There was no C-H stretching detected in the pure naringenin sample (wavenumbers 3,000 to 2,500 cm^−1^). The aromatic rings were visualised from 1,600 to 1,500 cm^−1^.

The −OH stretching, C-H stretching and aromatic rings from both chemicals, PVA and pure naringenin, were observed in Na/PVA nanoparticles sample (Fig. 2C). The −OH stretching was identified from wavenumbers of 4,000 cm^−1^ to 3,000 cm^−1^. End moiety infrared analysis of Na/PVA nanoparticles discloses consumption of the terminal C-H alkyne stretching at 3,275 cm^−1^ (Corley *et al*. 2019), with a shift to another alkenic moiety or alkane at 2,882 cm^−1^ (Takahama *et al*. 2013). The interaction of naringenin and PVA functional groups has tremendously narrowed the peak of C-H stretching. The FTIR spectrum for nanoparticles of naringenin showed no significant changes in absorption. The result signified the integration efficiency and stability of naringenin in PVA.

### The Release Property of Naringenin

**Figure 3:**
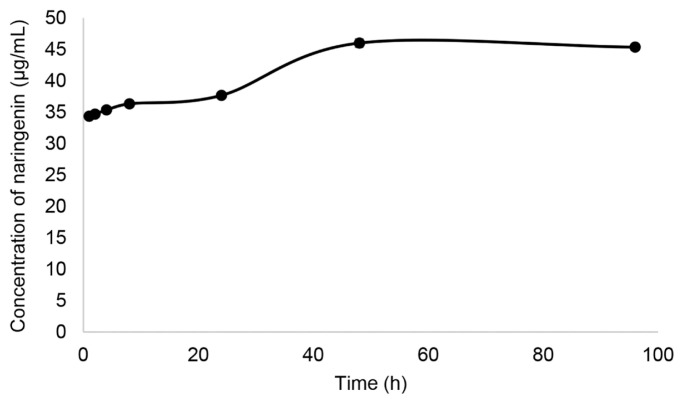
Naringenin release behaviour from Na/PVA nanoparticles which was observed for 96 h.

The release of naringenin from Na/PVA nanoparticles substance was monitored for 96 h (Fig. 3). Overall, there was an initial burst of the test drug at the first hour of the experimental period. The initial burst release effect is due to release of narigenin present on the surface of PVA nanoparticles. Naringenin was gradually released from Na/PVA nanoparticles substance starting from the beginning of the experimental period. From 0–48 h, the release followed the first order of kinetic, with the release rate of 0.87 μg h^−1^. The release was stagnant at 48 h, with a total release of 46 ± 0.7 μg mL^−1^.

### Antibacterial Efficiency: Disc Diffusion and Broth Microdilution Analyses

Several foodborne bacteria, i.e., *B. cereus*, *E. coli*, MRSA, *S. aureus*, *S. typhi* and *Yersinia* sp. Were selected to evaluate the antibacterial efficiency of Na/PVA nanoparticles on disc diffusion analysis. Out of six bacteria, only five bacteria were inhibited by Na/PVA nanoparticles substance (Table 1). The largest inhibition zone derived from discs, that of 14 mm, was displayed by MRSA. It was tailed by *S. aureus* with 11 mm and other bacteria was 10 mm each. Contrarily, *E. coli* did not exhibit any inhibition sign during the application of Na/PVA nanoparticles. The similar occurrence was obtained during the application of pure naringenin. *E. coli* is resistant to Na/PVA nanoparticles and pure naringenin. Overall, the Na/PVA nanoparticles showed better inhibitory activity than pure naringenin. It proves that the nano technology improves the antibacterial efficiency of the test substance.

Table 2 denotes the MIC and MBC of Na/PVA nanoparticles against the test bacteria. By comparing with the control, Na/PVA nanoparticles demonstrated an excellent potential to inhibit bacterial growth after 24 h of incubation. The lowest MIC value was recorded by *B. cereus* with 25 μg mL^−1^ and the highest value by *S. typhi* (100 μg mL^−1^). It is worth mentioning the MIC and MBC values for MRSA, *S. typhi* and *Yersinia* sp. were similar. To kill *B*. cereus, *S. aureus* and *S. typhi*, a higher concentration of the developed nanoparticles was needed as both bacteria were totally killed at a concentration of 100 μg mL^−1^. Overall, the MBCs, especially for *B. cereus* and *S. aureus*, were significantly greater than MICs. Thus, it denotes that the antibacterial efficiency of Na/PVA nanoparticles depends on the concentration. The antibacterial activity of the nanoparticles was concentration-dependent.

### Application of Na/PVA on Food Model

**Figure 4:**
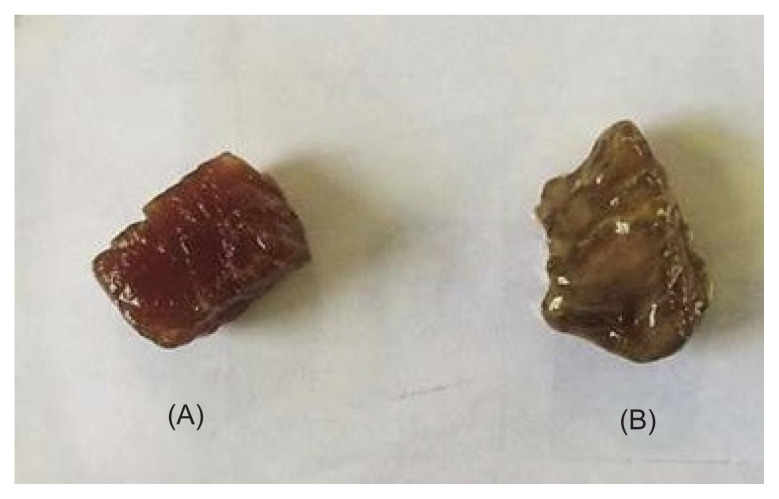
Visual appearance of meat at day 5 of experimental period. (A) was coated with Na/PVA nanoparticles and (B) was the uncoated sample.

**Figure 5:**
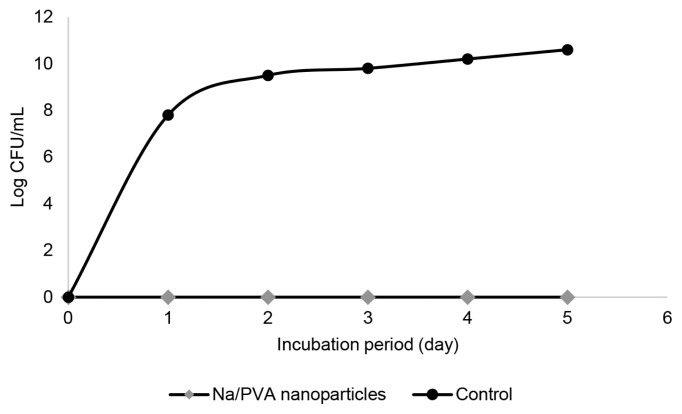
Bacterial load evaluation on meat with and without Na/PVA nanoparticles coating.

The meat sample coated with Na/PVA nanoparticles was physically fresher than the control meat even after five days of experimental period (Fig. 4). The meat was intact and maintained its redness. Contrarily, the control meat colour became pale, and a slime layer started to appear on the outer surface. An unattractive odour was also detected. These indicators signified spoilt meat. To further confirm our results, bacterial load analysis was conducted. Fig. 5 shows the antibacterial activity of Na/PVA nanoparticles, represented by the bacterial load in meat. As observed, the bacterial load of the control sample (without Na/PVA nanoparticles) was gradually augmented from day 0 to day 5. Besides, there was a distinctive difference between the control and the plates containing Na/PVA nanoparticles substance (*p* < 0.05). No apparent cells count perceived from the Na/PVA plates, which showed a 100% reduction of the bacterial load on the meat. The coating has notably reduced the bacterial load on the meat sample and indirectly could improve the shelf life of the test meat.

## DISCUSSION

Microbiological alterations account for the largest losses of food. The activities of foodborne microorganisms are aimed to reduce the shelf life of foodstuffs and impact consumers’ health. Thus, antimicrobial films and coatings are two active concepts applied in food packaging to eliminate, prevent and decelerate the growth of microorganisms on the food surface (Appendini & Hotchkiss 2002). In this study, a naringenin-grafted nanoparticles delivery system using PVA as a carrier was successfully developed. PVA is a FDA-approved chemical, comestible, non-toxic and eco-friendly biopolymer. It comprises an excellent chemical resistance and mechanical characteristics (Zanela *et al*. 2018). It is utilised in food packaging as a component of coatings and films (Gómez-Aldapa *et al*. 2020).

The successful development of the nanoparticles was determined through microscopy observation. Based on the TEM micrograph, the diameter of Na/PVA globules was less than 200 nm. This observation was comparable with recorded nanoparticles of naringenin by Joshi *et al*. (2018), Gaba *et al*. (2019) and Fuster *et al*. (2020). The homogeneous size dispersal obtained was due to the addition of Pluronic F-127 as a capping agent. It is an amphiphilic molecule that holds hydrophilic and lipophilic properties. This event indirectly provides better particles swelling in the water (Fuster *et al*. 2020). However, a low percentage of encapsulation efficiency (11.08%) was reported in this study. A higher encapsulation efficiency was reported by Sahadan *et al*. (2019). A low encapsulation percentage obtained during the synthesis of Na/PVA nanoparticles can be overcome by implementing the ionic watery, high-power ultrasounds and rapid desolvation in the solvent, prior to naringenin adsorption (Fuster *et al*. 2020). Besides, a low stirring speed during nanoparticle synthesis could affect the efficiency during the synthesis of nanoparticles. Li *et al*. (2017) stated that high shear stress could maximise the encapsulation efficiency by allowing the dispersing of viscous beads. Therefore, the encapsulation efficiency of naringenin could be improved by increasing the stirring speed during nanoparticle synthesis.

Besides, the interaction of naringenin and PVA functional groups was evidenced in the IR spectra. It can be concluded that the Na/PVA nanoparticles comprised −OH stretching, C-H stretching and aromatics ring. The disappearance of the absorption intensities of naringenin indicates that the functional groups of most of the drug’s molecules were successfully encapsulated in PVA, in the form of nanoparticles. In a previous study, Moreira *et al*. (2020) successfully proved that nanotechnology system applied on naringenin has modified its physicochemical profile and improvised drug release. According to their research, naringenin was loaded into the PVA gel using a solvent sorption technique. Among other simultaneous techniques, solvent sorption is a promising technique for drug integration during polymerisation. The unreacted substances can be eliminated, prior to drug loading. Besides, the loading amount of the PVA gel can be balanced by controlling the drug’s concentration, as well as swelling’s degree (Byun & Hong 2008). At the beginning of experiment, an initial burst release effect was observed. This property is crucial to ensure that sufficient amount of naringenin can be released to the food system, in order to achieve desirable antimicrobial effect. After that, the naringenin was released in a constant manner from Na/PVA nanoparticles. This manner represented the PVA as a good polymer that can amalgamate well with the naringenin. A swelling-controlled system has driven the release of naringenin since water and the hydrophilic polymer were involved. This statement was in consensus with Abu-Thabit and Makhlouf (2018). Hydrophilic polymers often encompass swelling-controlled systems. Once contact with water, the polymer network is not in dense condition and the drug disperses out due to unrestricted mobility of macromolecules, as well as the increasing volume of the system. Both PVA and Pluronic F127 have hydrophilic properties and excellent drug release characteristics (Jain *et al*. 2011). In short, PVA is an ideal polymer matrix for naringenin, as it produces an effective and sustainable delivery system for naringenin.

Naringenin is an aglycone portion of naringin. Previously, Ng *et al*. (2019) reported that naringenin synthesised by *Saccharomyces cerevisiae* can be potentially used as a natural food preservative. The compounds displayed an inhibitory effect on *S. aureus*. The data obtained from this study is in agreement with their study. The antimicrobial efficiency of naringenin-based samples was supported by Andrade *et al*. (2010), Lee *et al*. (2013), Agus *et al*. (2017), Teng, Li, *et al*. (2018) and Bonin *et al*. (2020). Andrade *et al*. (2010) declared that the fractions containing naringenin from the flowers of *Acacia podalyriifolia* inhibited both Gram-positive and Gram-negative bacteria such as *S. aureus*, *Staphylococcus epidermis*, *Streptococcus pyogenes*, *Klebsiella pneumoniae* and *Proteus mirabilis*. Agus *et al*. (2017) also reported that the naringenin-rich extract of pigeon pea leaves has exceptionally retarded the growth of *S. thypi*. A later investigation by Lee *et al*. (2013) has proven the antibacterial activity of naringenin against MRSA. It is also worth mentioning a report by Bonin *et al*. (2020) on *Baccharis dracunculifolia* extract, which inhibited both *B. cereus*, *S. aureus* and *Bacillus subtilis*. Once this extract was subjected to ultra high-performance liquid chromatography-high resolution mass spectrometry (UHPLC-HRMS), naringenin was recognised as one of the major components. In food industry, naringenin is widely used to reduce the advanced glycation end products in breadcrust. Furthermore, it improves the antioxidant and antibacterial activities of the bread without detrimental changes to the quality (Teng, Li, *et al*. 2018).

To date, naringenin-loaded nanoparticles system is widely explored, especially in pharmaceutical industries. Yen *et al*. (2009) developed a new naringenin-loaded nanoparticles delivery system via nanoprecipitation method by using PVA and Eudragit as carriers. The system successfully enhanced the bioavailability and hepatoprotective efficiency in mice, compared to pure naringenin. According to Fuster *et al*. (2020), the naringenin-loaded nanoparticles are potent anticancer agent. In their study, naringenin is integrated into silk fibroin to improve the therapeutic efficiency. These characteristics confer the silk fibroin as a potential candidate in controlling drug release (Fuster *et al*. 2020). Thus, selecting a suitable polymeric delivery carrier is crucial to validate the release of drugs in a controlled manner.

In this study, Gram-positive bacteria were more susceptible to the nanoparticles, compared to Gram-negative bacteria. The MICs and MBCs for the Gram-positive bacteria were ranged from 25–50 μg mL^−1^ and 50–100 μg mL^−1^, respectively. In contrast, a higher concentration value, that of > 50 μg mL^−1^, was needed to inhibit and kill the Gram-negative bacteria. A moderate rate of diffusion released from the test disc is one factor that hinders bacterial growth. A similar observation was reported by Fuster *et al*. (2020), where the application of naringenin-loaded silk nanoparticles in cancer therapy was limited because of its low water solubility and decelerate dissolution rate. These problems cause poor bioavailability at the targeted tumour spot. The same observation may apply on the antimicrobial activity of naringenin nanoparticles. Besides, the Gram-positive bacteria have a thicker peptidoglycan layer, that of 20–80 mm thickness, than the Gram-negative bacteria (Mai-Prochnow *et al*. 2016). In this study, the developed nanoparticles might favour in attacking the peptidoglycan cell wall of the Gram-positive bacteria. Due to this event, the bacterial cells become mechanically unstable and lysed. As for the Gram-negative bacteria, it owns dual-layer membranes built from peptidoglycan and lipopolysaccharide complexes (Silhavy *et al*. 2010). The bacterium like *E. coli* used this mechanism to resist the effect of Na/PVA nanoparticles, by limiting the penetration of nanoparticles to the cells *via* the complex cell wall.

Fresh beef is a protein source with high visual appeal and nutritional values, however, they are very susceptible to microbial contamination storage period. The treatment with Na/PVA nanoparticles maintained the visual qualities of fresh beef. Meat colour is an important factor that affects the visual appearance of fresh beef. By improving the microbiological quality of the fresh beef, the meat colour also improved. Besides, bacterial load is a significant factor that causes food spoilage. Therefore, it could be applied as an indicator of the freshness and quality of food. Fresh beef can be contaminated at several points, an exposed surface, high moisture and nutrient availability, as well as greater oxygen penetration. Microbial growth on beef can be detected via changes in colour, texture, bad odour and slime layer (Tsigarida & Nychas 2001). In this experiment, the bacterial load was significantly higher on uncoated meat (control) after 24 h of exposure, compared the beef sample coated with Na/PVA nanoparticles. There are two stages involved in bacterial attachment on meat surface. The first stage is started with poorly reversible sorption, which correlates with the interaction of intermolecular forces between molecules or other physicochemical variables. The second stage involved the production of extracellular polysaccharides (EPS), which is identified as an irreversible attachment with surfaces (Erkmen & Bozoglu 2016). The EPS extension leads to the formation of a biofilm matrix around the meat. The existence of the matrix has made the bacteria tolerable to extreme conditions and resistant to antibiotic agents. A 100% reduction of bacterial load signified the antibacterial efficiency of Na/PVA nanoparticles. Thus, this finding has identified the Na/PVA nanoparticles as a potential meat preservative, which improve the organoleptic properties of fresh beef.

## CONCLUSION

In this study, Na/PVA nanoparticles were successfully synthesised based on TEM micrograph. The nanoparticles exhibited broad spectrum antimicrobial activity, which inhibited both Gram-positive and Gram-negative foodborne. Besides, the fresh beef coated with Na/PVA nanoparticles displayed a 100% reduction in bacterial load. The coating of nanoparticles significantly prolonged the shelf life of fresh beef. Thus, the developed nanoparticles can be potentially used nano-preservative to maintain the microbiological quality of fresh beef.

## Figures and Tables

**Table 1:** Antibacterial efficacy of Na/PVA nanoparticles against foodborne bacteria on disc diffusion assay.

Foodborne microorganism	Diameter of inhibition zone (mm)			

	Control	Na/PVA nanoparticles	Blank nanoparticles	Pure-naringenin
*B. cereus*	–	10 ± 0.2	–	9 ± 0.2
*E. coli*	–	–	–	–
MRSA	–	14 ± 0.1	–	8 ± 0.2
*S. aureus*	–	11 ± 0.1	–	7 ± 0.2
*S. thypi*	–	10 ± 0.2	–	8 ± 0.2
*Yersinia* sp.	–	10 ± 0.2	–	8 ± 0.2

**Table 2:** MIC and MBC values of Na/PVA nanoparticles tested on bacteria.

Foodborne microorganism	Na/PVA nanoparticles	

	MIC (μg mL^−1^)	MBC (μg mL^−1^)
*B. cereus*	25	100
MRSA	50	50
*S. aureus*	50	100
*S. thypi*	100	100
*Yersinia* sp.	50	50
